# Interventional Optical Imaging-Monitored Synergistic Effect of Radio-Frequency Hyperthermia and Oncolytic Immunotherapy

**DOI:** 10.3389/fonc.2021.821838

**Published:** 2022-01-24

**Authors:** Hui Zheng, Feng Zhang, Wayne Monsky, Hongxiu Ji, Weizhu Yang, Xiaoming Yang

**Affiliations:** ^1^ Image-Guided Biomolecular Intervention Research and Division of Interventional Radiology, Department of Radiology, University of Washington School of Medicine, Seattle, WA, United States; ^2^ Department of Interventional Radiology, Fujian Medical University Union Hospital, Fuzhou, China; ^3^ Department of Pathology, Overlake Medical Center and Incyte Diagnostics, Bellevue, WA, United States

**Keywords:** indocyanine green, interventional optical imaging, oncolytic immunotherapy, radiofrequency ablation, radiofrequency hyperthermia

## Abstract

**Purpose:**

To develop a new interventional oncology technique using indocyanine green (ICG)-based interventional optical imaging (OI) to monitor the synergistic effect of radiofrequency hyperthermia (RFH)-enhanced oncolytic immunotherapy.

**Materials and Methods:**

This study included (1) optimization of ICG dose and detection time-window for intracellular uptake by VX2 tumor cells; (2) *in-vitro* confirmation of capability of using ICG-based OI to assess efficacy of RFH-enhanced oncolytic therapy (LTX-401) for VX2 cells; and (3) *in-vivo* validation of the interventional OI-monitored, intratumoral RFH-enhanced oncolytic immunotherapy using rabbit models with orthotopic liver VX2 tumors. Both *in-vitro* and *in-vivo* experiments were divided into four study groups (n=6/group) with different treatments: (1) combination therapy of RFH+LTX-401; (2) RFH alone at 42°C for 30 min; (3) oncolytic therapy with LTX-401; and (4) control with saline. For *in-vivo* validation, orthotopic hepatic VX2 tumors were treated using a new multi-functional perfusion-thermal radiofrequency ablation electrode, which enabled simultaneous delivery of both LTX-401 and RFH within the tumor and at the tumor margins.

**Results:**

In *in-vitro* experiments, taking up of ICG by VX2 cells was linearly increased from 0 μg/mL to 100 μg/mL, while ICG-signal intensity (SI) reached the peak at 24 hours. MTS assay and apoptosis analysis demonstrated the lowest cell viability and highest apoptosis in combination therapy, compared to three monotherapies (*P*<0.005). In *in-vivo* experiments, ultrasound imaging detected the smallest relative tumor volume for the combination therapy, compared to other monotherapies (*P*<0.005). In both *in-vitro* and *in-vivo* experiments, ICG-based interventional optical imaging detected a significantly decreased SI in combination therapy (*P*<0.005), which was confirmed by the “gold standard” optical/X-ray imaging (*P*<0.05). Pathologic/laboratory examinations further confirmed the significantly decreased cell proliferation with Ki-67 staining, significantly increased apoptotic index with TUNEL assay, and significantly increased quantities of CD8 and CD80 positive cells with immunostaining in the combination therapy group, compared to other three control groups (*P*<0.005).

**Conclusions:**

We present a new interventional oncology technique, interventional optical imaging-monitored RFH-enhanced oncolytic immunotherapy, which may open new avenues to effectively manage those patients with larger, irregular and unresectable malignancies, not only in liver but also the possibility in other organs.

## Introduction

Advanced techniques of interventional oncology, including tumor ablation and embolization, have become important tools in managing patients with primary and secondary malignancies. These efficacious interventional therapies have prominent advantages, including being minimally invasive and repeatable, offering low risk of complications, and requiring only a short hospital stay. Among different interventional tumor ablation techniques, radiofrequency ablation (RFA) is universally accepted, well studied, and the recommended interventional technique for eradicating small (< 3 cm) lesions, with equal overall survival, safety and cost-effectiveness as microwave ablation (MWA) (1, 2). However, for ablation of larger and irregular lesions, these ablations appear to be limited by incomplete tumor killing, leaving residual tumor at the tumor margin/periphery (36.5%) (3, 4). Ultimately, residual viable tumor cells at the tumor margin/periphery result in residual tumor and recurrence, leading to treatment failure (4, 5).

Several neoadjuvant or adjuvant approaches have been tested to overcome these limitations of thermal ablation (6). One example is to administer chemoembolization prior to RFA, which reduces downstream hepatic arterial blood flow and thereby diminishes the “heat-sink” effect (7). However, this combination requires two consecutive treatment sessions, which poses additional operational risks, longer hospital stays and higher hospital costs for patients. Another example of combination therapy is to apply systemic chemotherapy in addition to RFA. This bears the limitations of systemic chemotherapy, i.e., less than adequate therapeutic drug dose reaching the tumor site, toxicity to other vital organs, and, most importantly, frequent development of multi-drug resistance.

Immunotherapy has become one of the frontiers in modern medical oncology (8–11). Oncolytic cancer therapy represents a new promising strategy in the immunotherapy, with its mechanisms of reducing local immunosuppression, reinstating and enhancing systemic anticancer T-cell functions, which thus mediates abscopal effects and hence long-term protection from metastatic relapse (10, 11). Oncolytic cancer therapy employs microbial- or non-microbial based oncolytic therapeutics. Among different microbial- or non-microbial oncolytic therapeutics, LTX-401, a cationic amphiphilic peptide derivative, is currently recognized as the first-in-class, more advanced oncolytic therapeutic (12, 13). LTX-401 can locally stimulate long-term anticancer immune responses, with high effectiveness against a broad panel of both drug-resistant and drug-sensitive cancers (14–16). Immunogenic cell death (ICD) can attract dendritic cell precursors to the proximity of dying cancer cells and stimulate the uptake of dead cell-associated antigens followed by optimal tumor antigen cross-presentation to stimulate cytotoxic T lymphocyte responses (17, 18). LTX-401 induces necrotic cancer cell death followed by the release of damage-associated molecular pattern molecules (DAMPs), which are the molecular markers of ICD (12).

Recent studies from our group and others have successfully confirmed that image-guided interventional radiofrequency hyperthermia (RFH, at a sub-lethal temperature <60°C) can greatly enhance chemo- and gene therapies of malignancies in various organs (19–21). The recognized mechanisms of RFH-enhanced therapies include tissue fracture *via* heating, increased permeability of cytoplasmic membranes, disruption of cellular metabolism, activation of membrane associated pumps, and activation of the heat shock protein pathway (22–24). These mechanisms effectively facilitate the entrance of therapeutic agents into target tumor cells, and thereby promote the destruction of tumor tissue. Based on these findings we sought to develop a new approach, to simultaneously deliver oncolytic therapeutics to the tumor periphery during thermal ablation. This novel concept combines RFA-associated RFH, i.e., combining usual thermal ablation with lethal heat (>60°C) destroying tumor cells in the tumor center, with peri-tumoral specific delivery of high-dose oncolytic therapeutics to further kill tumor cells within the difficult to treat periphery of the ablated tumors (24, 25). Thus, this innovative synergistic combination could ensure complete eradication of all viable tumor cells in and adjacent to the tumor being treated, while sparing adjacent normal structures.

Optical imaging (OI) refers to a variety of techniques using either near-infrared (NIR-I & NIR-II, 700–1700 nm)/short-wave infrared (SWIR, 1000–2000 nm) or visible (400–700 nm) light, to provide molecular, morphologic, and functional information, probing absorption, scattering, and fluorescence properties of cells or tissues (26). The advantages of OI include real-time imaging capability, cost-effective, portable, non-ionizing, and generally well tolerated by patients (26, 27). Application of optical imaging dyes or contrast agent, such as FDA-approved indocyanine green (ICG), has further enhanced the usefulness of optical imaging techniques, providing better detection of relatively deep-seated lesions and better guidance of cancer treatments (28, 29). However, the OI for detection of deep-seated lesions is challenging, since the tissue penetration depth of current ICG-based fluorescence imaging is approximately 1 cm (30, 31). To solve this problem of the limited penetration depth with current OI, we have recently established a new image-guided interventional OI approach, which are capable of precisely guiding the percutaneous position of micro-OI detectors into the targets, avoiding tissue scattering and reflection along the pathway of OI light (32, 33).

In this study, we attempted to specifically address the clinical problem of post-ablation tumor recurrence, by fully integrating the advantages of these approaches, including image-guided interventional oncology, OI, and hyperthermia-enhanced oncolytic immunotherapy, with our final goal to develop a completely new, “one-stop-shop” interventional oncologic technique, named “interventional OI-monitored RFH-enhanced direct oncolytic cancer therapy”.

## Materials and Methods

### Study Design

The present study was carried out in two phases: (a) *in-vitro* experiments to confirm RFH-enhanced oncolytic immunotherapeutic efficacy of LTX-401 on VX2 tumor cells; and (b) *in-vivo* technical feasibility validation using ICG-based OI to monitor RFH-enhanced LTX-401 oncolytic immunotherapeutic efficacy on rabbit models with the same orthotopic hepatic VX2 tumors.

For both *in-vitro* experiments with VX2 tumor cells and *in-vivo* experiment using rabbit orthotopic VX2 hepatic tumors, the cells and animals were divided into four groups of (1) combination therapy of RFH+LTX-401; (2) RFH alone at 42°C for 30 minutes; (3) oncolytic therapy with LTX-401; and (4) control with saline (n=6/group).

### *In-Vitro* Confirmation

#### Cell Culture

VX2 tumor cells (IDAC, Tohoku University, Japan) were seeded (8×10^4^ per well) in four chamber cell culture slides (Thermo Fisher Scientific, Rochester, NH) and maintained in RPMI 1640 Medium supplemented with 10% fetal bovine serum (Gibco, Grand Island, NY), and incubated at 37°C with a 5% carbon dioxide atmosphere.

### Optimizing ICG Dose and Time-Window for Uptake by Rabbit VX2 Cancer Cells

VX2 cells seeded (8×10^4^ per well) in four chamber cell culture slides treated with ICG (Patheon Italia S.P.A, Ferentino, Italy) at (i) concentrations of 0, 25, 50, 75, 100, 125 μg/mL for 24 hours; (ii) a concentration of 100 μg/mL with various incubation times of 0, 2, 6, 12, 24, and 48 hours. ICG-incubated cells were washed twice with phosphate buffered saline (PBS) to remove the free ICG, fixed with 4% paraformaldehyde, dried at room temperature, counterstained with 4’, 6-diamidino-2-phenylindole (DAPI; SouthernBiotech, Birmingham, AL), and then imaged with a fluorescence microscope (Excitation: FF01-769/41-25, Emission: FF01-832/37-25; Semrock, Rochester, NY).

For optimizing ICG concentration and incubation time, 3×10^5^ ICG-treated cells were collected and resuspended in 0.6-mL microcentrifuge tubes (Fisherbrand^®^, Thermo Fisher Scientific Inc.) with 0.2-mL cell culture medium. Subsequently, the cell-containing tubes were imaged by the optical/X-ray imaging system (*In-vivo* Xtreme; Bruker, Billerica, MA) at the excitation wavelength of 760 nm, emission wavelength of 830 nm, field of view of 120 × 120 mm, and exposure time of 1 minute. The fluorescence signal intensities (SIs) of cells in tubes were measured using the Bruker molecular imaging software.

### The Half Maximal Inhibitory Concentration (IC50) of LTX-401

MTS assay [(3-(4,5-dimethylthiazol-2-yl)-5-(3-carboxy-methoxyphenyl)-2-(4-sulfophenyl)-2H-tetrazoliu)] (Promega Corpora, Madison, WI) was used to evaluate the cytotoxicity of LTX-401 (C₂₃H₃₅Cl₂N₃O, MedChemExpress, Monmouth Junction, NJ) in VX2 cancer cells. 8×10^4^ cells were seeded in 96-Well Plate (Becton Dickinson Labware), incubated with LTX-401 at the concentrations of 0, 5, 10, 15, 20, 30, 40, 50, 70, and 90 μM for 4 hours. The cells were washed with serum-free RPMI 1640 and incubated with MTS solution for 2 hours according to the manufacturer’s protocol. The absorbances were measured at 490 nm with a 1420 Multilabel Counter (VICTOR^3^_TM_ V, PerkinElmer, Singapore).

### RFH-Enhanced Killing Effect of LTX-401

RFH was performed by placing a 0.022-inch RF heating wire under the bottom of the four-chamber slides in 37°C water bath. A 400-μm fiber optical temperature probe (PhotonControl, Burnaby, British Columbia, Canada) was placed in the chamber for temperature monitoring. By adjusting RF output power to maintain the temperature of the chamber four at 42 ± 1°C, while the chamber one remained at 37°C.

### Cell Proliferation Assay

Cells proliferation was evaluated by MTS assay after the treatments. Relative cell proliferations of different cell groups were calculated by using the equation of A_treated_-A_blank_/A_control_-A_blank_, where A is absorbance. After the treatments, cells were treated with ICG at concentrations of 100 μg/mL for 24 hours. ICG-cells were washed twice with PBS to remove free ICG, fixed with 4% paraformaldehyde, and then dried at room temperature. The cells were counterstained with DAPI, and then imaged with the fluorescence microscope at the parameter of described above.

### Apoptosis Assay

The percentages of viable as well as apoptotic cells were quantified by flow cytometry. Cells were stained with AnnexinV-fluorescein isothiocyanate and 7-Amino-Actinomycin D (7-AAD) (BD Biosciences, San Diego, CA) in a binding buffer along with the appropriate control. Total number of Annexin V and 7-AAD positive cells were counted using the flow cytometer. The data was analyzed using FloJo Data Analysis software.

### *In-Vitro* Interventional Optical Imaging of Treated Cells

**Figure 1:**
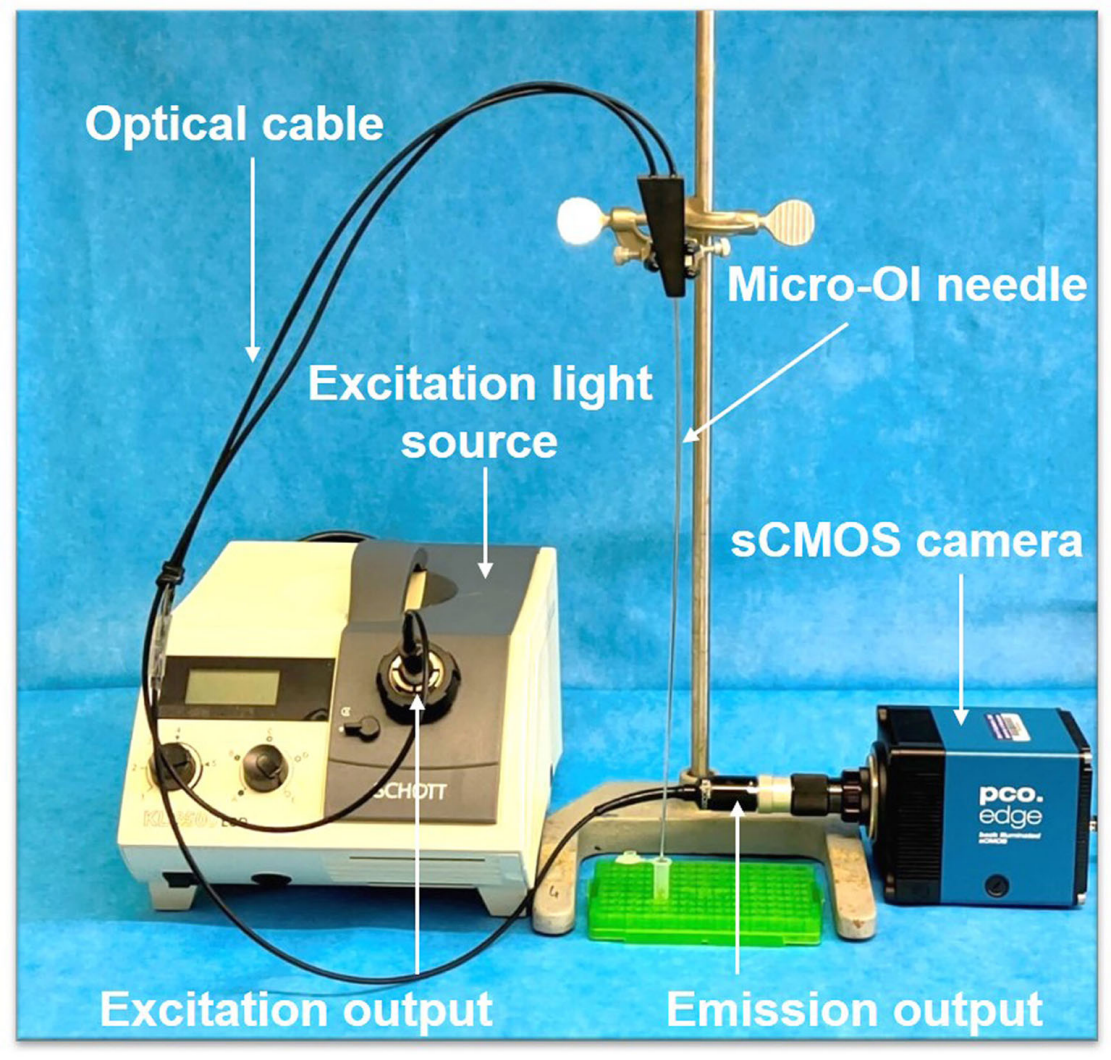
The interventional optical imaging (OI) system. The interventional OI system is constructed with a micro-OI needle and a sCMOS camera that is connected to an excitation light source and a personal computer *via* its emission output.

The interventional OI system was assembled as presented in **Figure 1**. A 17-gauge micro-OI needle was illuminated by a 250-W halogen light source (KL 2500 LCD, Schott, Germany) that was equipped with an excitation filter (ET775/50x, Chroma Technology Corp., Bellows Falls, VT). The NIR fluorescence signals of ICG in cells were acquired with a metal-oxide-semiconductor (sCMOS) camera (Pco.edge 4.2 bi; PCO AG, Kelheim, Germany) through a band pass emission filter (ET845/55m, Chroma Technology Corp.). The sCMOS camera was connected to a personal computer for imaging acquisition and storage, at the resolution of 2048×2048, field of view of 1.1×1.1 mm, and pixel size of 6.5×6.5 μm^2^ over an exposure time of 2 seconds.

The *in-vitro* interventional optical images of ICG-treated VX2 cells (ICG-cells) were performed using the optimized protocol (ICG concentration at 100 μg/mL and incubation duration of 24 hours). Cells in four groups were rinsed twice with PBS, trypsinized, centrifuged, and then 3×10^5^ cells were resuspended in 0.6-mL microcentrifuge tubes (Fisherbrand^®^) with 0.2-mL cell culture medium. The optical images were obtained by inserting the micro-OI needle into the sample in a dark environment. The optical images were analyzed using the Bruker molecular imaging software.

### Optical/X-Ray Imaging of Treated Cells

1.5×10^5^ ICG-VX2 cells were resuspended in a 96-well plate with 0.1-mL cell culture medium. Subsequently, the cell-containing plate was imaged with the optical/X-ray imaging system, followed by comparing fluorescence SIs among different cell groups, as described above.

### *In-Vivo* Validation

#### Creation of Liver VX2 Tumors in Rabbits

Animal experiments were conducted according to institutional guidelines and prior IACUC approval. To create orthotopic hepatic VX2 tumors in recipient rabbits, a 3–4 cm-long incision was made below the sub-xiphoid process to expose the left lobe of the liver. Then 3-5 1-2 mm^3^ tissue fragments from the donor VX2 tumor was directly implanted into the subcapsular parenchyma of the left liver lobe, followed by 5-minute compression of the tumor implantation site with a gelatin sponge (Pharmacia & Upjohn Co, Kalamazoo, MI), and the closure of the abdominal incision with layered sutures.

### Treatment of Liver VX2 Tumors

Almost 2 weeks after tumor implantation, twenty-four adult female New Zealand White rabbits, weighing 2-3 kg, with liver VX2 tumors were randomly allocated to four groups: (1) combination therapy of RFH+LTX-401; (2) RFH alone at 42°C for 30 minutes; (3) oncolytic therapy with LTX-401; and (4) saline as the control (n=6/group).

**Figure 2:**
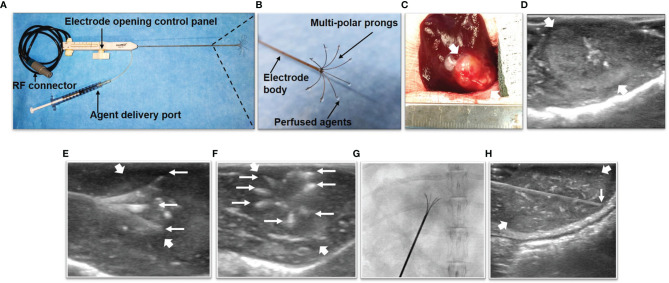
The multi-functional perfusion-thermal radiofrequency (RF) electrode, treatment process of liver tumor, and *in-vivo* interventional optical imaging process of liver tumor. **(A)** The multi-functional perfusion-thermal RF electrode with multipolar agent infusion prongs. Micro-thermosensors are equipped at the tips of the prongs for real-time measurement of RFH at approximately 42°C. **(B)** The details of RF electrode tip. **(C)** Morphology image of tumor (arrow) during surgery, which is detected by ultrasound imaging **(D)**. The perfusion-thermal electrode is advanced into the center of the tumor (between large arrows) under real-time ultrasound guidance. **(E)** Longitudinal and **(F)** transverse sonographic images, showing the electrode prongs (small arrows). The electrode prongs (small arrows) are opened to the desired size array, further confirmed by X-ray image **(G)**, to completely cover the tumor peripheral zone for sufficient delivery of the therapeutic agent. During delivery of LTX-401, RFH at the tumor is generated with the same device to further enhance oncolytic treatment. **(H)** Under real-time ultrasound imaging guidance, the micro-OI needle (small arrow) was advanced into the periphery of the tumor (between large arrows), to generate intratumoral “inside-output” optical images at different directions/points around the tumor periphery.

We used a multi-functional perfusion-thermal RF electrode, which has multiple prongs with the integrated thermal sensors, to simultaneously deliver RF-induced thermal energy and LTX-401 in tumors (**Figures 2A, B** *)*. *Via* laparotomy, the RF electrode was precisely positioned in the center of the tumor under real-time ultrasound imaging guidance and confirmed by X-ray imaging (**Figures 2C–G**). Then, LTX-401 (at 1-mg/kg bodyweight) was directly infused into liver tumors through the prongs of the perfusion-thermal RF electrode, followed by intratumoral RFH at 42°C for 30 minutes.

### Post-Treatment Follow-Up

Ultrasound imaging was used to follow up the tumor size changes at day 0, day 7, and day 14 after treatments. The axial (X) and longitudinal (Y) diameters of tumors, as well as tumor depths (Z) were measured on the ultrasound images at the maximal tumor dimensions. The volume of each tumor was calculated according to the equation: V= X*Y*Z*π/6, where V is tumor volume. Data was presented as relative tumor volume (RTV) by using the following equation: RTV = V_Dn_/V_D0_, where D0 is the day before treatments, and Dn is the day after the treatments.

### *In-Vivo* Interventional Optical Imaging of Liver VX2 Tumors

At day 14 after the treatments, ICG (0.5mg/kg bodyweight) was injected intravenously *via* the rabbit ear vein 24h before optical imaging at animal euthanization. Under real-time ultrasound imaging-guidance, the interventional micro-OI needle was inserted closed to the tumor, where the fluorescence signal of normal liver parenchyma was acquired. Then, the micro-OI needle was advanced into the tumor to achieve intratumoral “inside-output” optical imaging at six different positions of the ablated tumor periphery (at 3, 6, 9, 12 o’clock along the equator and two points at the north and south pole), to detect the ICG-emitting fluorescence of residual tumor (**Figure 2H**). Then, the fluorescence signals were analyzed, with data presented as signal-to-background ratio (SBR) by using the following equation: SBR = SI_T_/SI_L_, where T represents tumor, and L is liver.

### *Ex-Vivo* Optical/X-Ray Imaging of Liver VX2 Tumors

The tumors were harvested, and then imaged with the optical/X-ray imaging system with the same parameters described above. The SIs of fluorescence of different groups were measured and compared statistically. The *ex-vivo* OI of the gross and sectioned tumor specimens functioned as a “Gold standard” to corroborate the findings achieved by the *in-vivo* interventional OI system, with final confirmation by pathologic examination.

### Pathologic Correlation/Confirmation

Tumor tissues were fixed with 4% formalin in PBS for 24 hours, embedded in paraffin and then sliced into 4-μm sections for the following histopathological staining preparations: (a) hematoxylin-eosin (H&E) to confirm the formation of liver VX2 tumor; (b) Ki-67 immunostaining (Epredia, Kalamazoo, MI) to assess tumor proliferations among four groups; and (c) terminal deoxynucleotidyl transferase biotin-dUPT nick end labeling (TUNEL, EMD Millipore Corporation, Temecula, CA) to determine tumor cell apoptosis. In addition, to evaluate immunotherapeutic effects, immunostaining for (d) CD8 (Abcam, Cambridge, MA) and (e) CD80 (LSBio, Seattle, WA) were carried out. Positive cells were imaged using a microscope, and semi-quantified by Image-pro Plus 6.0 software (Media Cybernetics, Silver Spring, MD).

### Statistical Analysis

Statistical software (SPSS, Version 19.0; Chicago, III) was used for all data analyses. The non-parametric Mann-Whitney U test was used to compare the differences among various cell and animal groups with four different treatments. A *P* value of less than 0.05 was considered significant.

## Results

### *In-Vitro* Optimization of ICG Dose and Time-Window for OI of ICG-Cells

**Figure 3:**
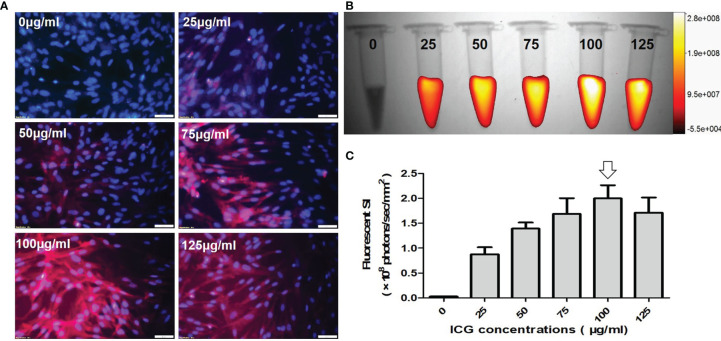
Optimization of indocyanine green (ICG) dose for *in-vitro* optical imaging of ICG-treated VX2 tumor cells. **(A)** Fluorescence microscopic images of VX2 cells labeled at different ICG concentrations from 0 μg/ml to 125 μg/ml, showing that ICG-emitting pink fluorescent signals become more intense as ICG concentration increases (Scale bars, 50 μm). **(B, C)** Quantitative optical/X-ray images further demonstrate the highest fluorescent SI at 100 μg/ml (arrow) for sufficient ICG uptake by VX2 cells.

Fluorescence microscopic images of VX2 cells incubated with ICG at different concentrations from 0 μg/ml to 125 μg/ml, showing that ICG-emitting pink fluorescent signals became more intense as ICG concentration increases (**Figure 3A**). Quantitative optical/X-ray images further demonstrated the fluorescence signal reached the plateau as the ICG concentration at 100 μg/ml (**Figures 3B, C**). Therefore, the optimal concentration ICG was determined at 100 μg/mL.

**Figure 4:**
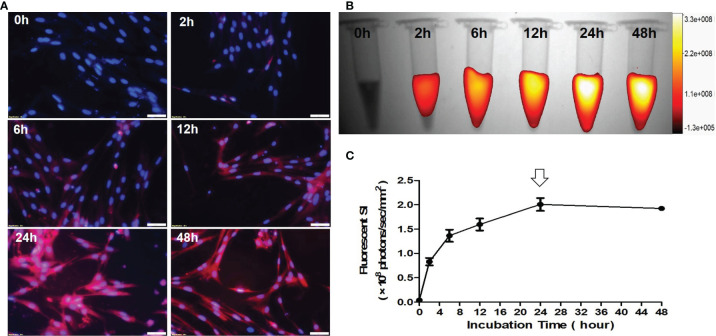
Optimization of the detection time-window for optical imaging of indocyanine green (ICG)-treated VX2 tumor cells. **(A)** Fluorescence microscopic images of VX2 cells labeled by ICG with different incubation times from 0 h to 48 h, displaying that ICG-emitting pink fluorescent signal becomes more intense as the incubation times increase (Scale bars, 50 μm). **(B, C)** Quantitative optical/X-ray images further demonstrate ICG SI becomes peek at 24 hours (arrow) as the optimum time window for sufficient ICG uptake by VX2 cells.

Fluorescence microscopic images of VX2 cells treated by ICG with different incubation durations from 0 h to 48 h, showing that ICG-fluorescence signal became more intense as the incubation times increase (**Figure 4A**). Quantitative optical/X-ray images further demonstrated the ICG fluorescence signal in VX2 cells reached the peak after 24 hours of incubation (**Figures 4B, C**). Thus, the optimal time point of 24 hours post treatment of VX2 cells with ICG was selected as the optimum parameter for the next *in-vitro* interventional optical imaging.

### *In-Vitro* Confirmation

**Figure 5:**
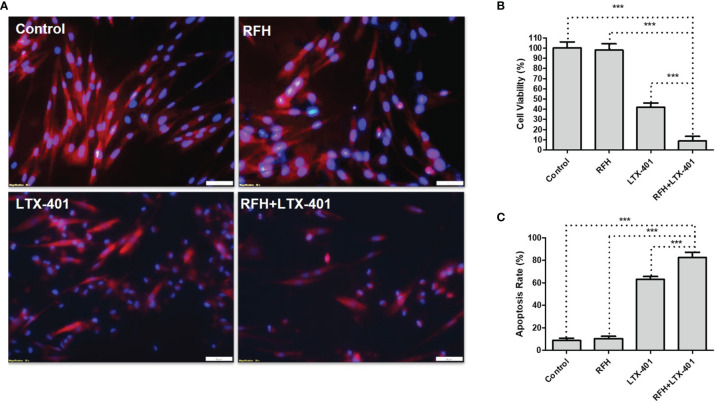
Fluorescence microscopic images, cell viability and apoptosis analysis. **(A)** Fluorescence microscopic images of different cell groups with various treatments, demonstrating a decrease of ICG signal (pink) and nuclear atrophy (blue), as well as the reduction in cell size with the combination therapy of RFH + LTX-401 (Scale bars, 50 μm). **(B)** MTS assay for evaluation of cell viability after different treatments, showing a significant decrease of cell viability with the combination therapy of RFH + LTX-401, compared to other treatments (***=*P*<0.005). **(C)** Annexin V-FITC/7-AAD flow cytometry for evaluation of cell apoptosis further demonstrates a significant highest apoptosis with the combination therapy of RFH + LTX-401, compared to other monotherapies (***=*P*<0.005).

**Figure 6:**
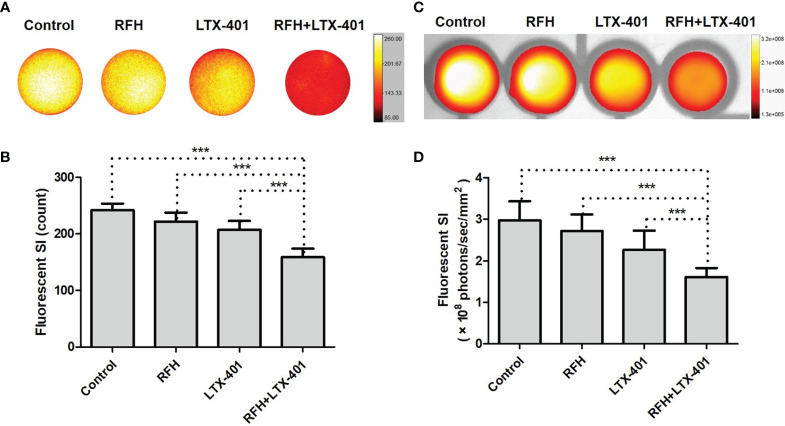
*In-vitro* interventional optical images and optical/X-ray images of different cell groups. **(A, B)** *In-vitro* interventional optical images demonstrate a significant decrease of ICG signal intensity (SI) with the combination therapy of RFH + LTX-401, compared to other treatments (***=*P*<0.005). **(C, D)** Optical/X-ray images further confirm the same results, a significant decrease of ICG SI with the combination therapy of RFH + LTX-401, compared to other treatments (***=*P*<0.05).

IC50 of LTX-401 was determined at 32.2 μM. Fluorescence microscopic images demonstrated the significant decrease of ICG signal, nuclear atrophy, as well as the reduction in cell size with the combination therapy of RFH+LTX-401 (**Figure 5A**). MTS assay demonstrated the significant decrease of cell viability with the combination therapy of RFH+LTX-401, compared to LTX-401 alone, RFH alone, and saline (8.72 ± 4.70% vs 41.73 ± 4.32% vs 97.93 ± 6.24% vs 100.01 ± 6.07%, respectively; *P*<0.005) (**Figure 5B**). Flow cytometry detected the highest apoptosis with the combination therapy, compared to other monotherapies (82.45 ± 4.79% vs 63.07 ± 2.59% vs 10.38 ± 2.03% vs 8.62 ± 2.10%, *P*<0.005) (**Figure 5C**). The *in-vitro* interventional optical images confirmed a significant decrease of ICG signals in cells with the combination therapy, compared to other monotherapies (158.58 ± 15.37 vs 207.21 ± 15.60 vs 221.89 ± 15.83 vs 241.55 ± 12.07 counts, *P*<0.005) (**Figures 6A, B**). Optical/X-ray images further confirmed a significant decrease of ICG SI with the combination therapy, compared to other monotherapies ((1.61 ± 0.22 vs 2.27 ± 0.46 vs 2.72 ± 0.40 vs 2.97 ± 0.46)×10^8^ photons/sec/mm^2^, *P*<0.05) (**Figures 6C, D**).

### *In-Vivo* Validation

**Figure 7:**
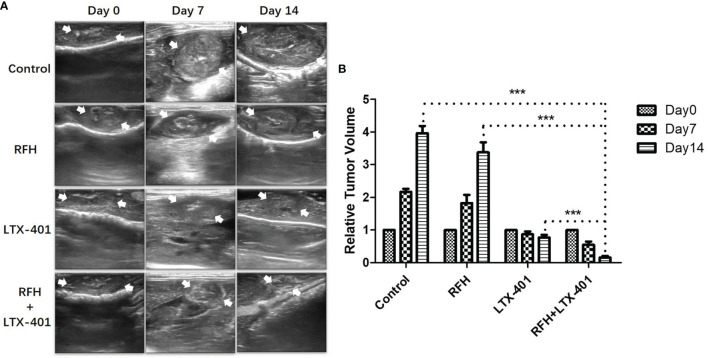
Ultrasound-imaging follow-up of different treatment groups. **(A, B)** Ultrasound images demonstrate significantly decreased sizes of the treated tumors (between arrows) at day 14 after the combination therapies with LTX-401 plus RFH, compared to other monotherapies (***=*P*<0.005).

**Figure 8:**
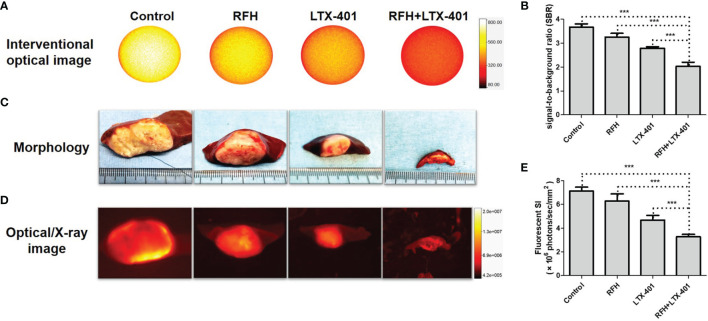
**|** *In-vivo* interventional optical imaging, confirmed by optical/x-ray imaging. **(A, B)** Interventional optical images show a significant decrease of ICG (yellow) signal-to-background ratio (SBR) with the combination therapy of RFH + LTX-401 (***=*P*<0.005). **(C)** Morphology demonstrates the smallest tumor size, and **(D, E)** Optical/X-ray images further confirm that combination therapies of oncolytic LTX-401 with RFH results in significant decrease of the average fluorescent SI of tumors in the combination therapy group, compared to the other three groups (***=*P*<0.005).

For *in-vivo* experiments, ultrasound images showed that combination therapies of oncolytic LTX-401 with RFH resulted in the decreases of average tumor volumes, compared to other monotherapies (0.16 ± 0.05 vs 0.77 ± 0.08 vs 3.38 ± 0.31 vs 3.96 ± 0.22%, *P*<0.005) (**Figures 7A, B**). Interventional optical image showed a significant decrease of ICG SBR with the combination therapy, compared to other treatments (2.03 ± 0.18 vs 2.79 ± 0.07 vs 3.26 ± 0.15 vs 3.68 ± 0.13, *P*<0.005) (**Figures 8A, B**). Morphology of sectioned tumor specimens demonstrated the smallest tumor size with the combination therapy (**Figure 8C**). The “gold standard” optical/X-ray images further confirmed that combination therapies of oncolytic LTX-401 with RFH caused decreases of the average fluorescent SI of tumors ((3.28 ± 0.20 vs 4.67 ± 0.40 vs 6.28 ± 0.61 vs 7.12 ± 0.34)×10^6^ photons/sec/mm^2^, *P*<0.005) (**Figures 8D, E**).

**Figure 9:**
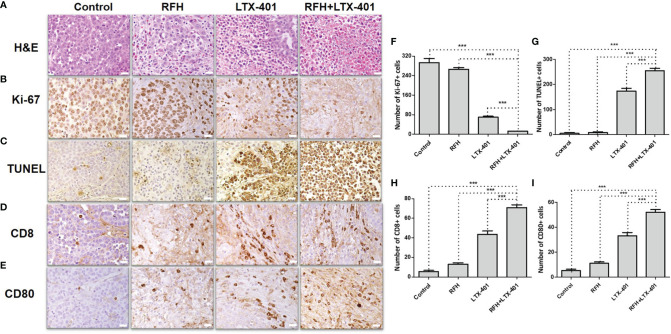
Pathology confirmation. **(A)** H&E images demonstrate a nuclear atrophy and hyperchromatism, as well as the reduction in cell size with the combination therapy of RFH + LTX-401. **(B, F)** Ki-67 staining shows a significant inhibition of cancer cell proliferation in the combination therapy group, as evidenced by the fewest number of brown-stained cells (***=*P*<0.005). **(C, G)** TUNEL assay further confirms significantly increased apoptotic index in combination therapy group, as evidenced by the highest number of brown-stained cells (***=*P*<0.005). **(D, H)** CD8 staining and **(E, I)** CD80 staining further demonstrate the highest number of brown-stained cells in combination therapy group (***=*P*<0.005), in comparison to other monotherapy groups. (Scale bars, 20 μm).

H&E images demonstrated a nuclear atrophy and hyperchromatism, as well as the reduction in cell size with the combination therapy (**Figure 9A**). The cell proliferation analysis by Ki-67 immunostaining displayed the lower proliferation activity in the combination therapy group, compared with other three monotherapies (12.45 ± 0.69 vs 69.63 ± 4.22 vs 266.20 ± 6.93 vs 293.63 ± 16.83, *P*<0.005) (**Figures 9B, F**). The TUNEL assay further confirmed significantly increased apoptotic index in combination therapy group (254.78 ± 9.49 vs 173.58 ± 10.44 vs 8.40 ± 2.15 vs 4.88 ± 3.08, *P*<0.005) (**Figures 9C, G**). CD8 staining showed the largest number of positive cells in combination therapy group (70.90 ± 2.81 vs 43.55 ± 3.42 vs 12.98 ± 1.50 vs 5.45 ± 1.35, *P*<0.005) (**Figures 9D, H**), while CD80 staining demonstrated the largest number of positive cells in combination therapy group, compare to other three groups (52.23 ± 2.01 vs 33.32 ± 2.56 vs 11.07 ± 1.16 vs 5.28 ± 1.12, *P*<0.005) (**Figures 9E, I**).

## Discussion

In this study, the results obtained through serial *in-vitro* confirmation and *in-vivo* validation experiments have confirmed the synergistic effect of interventional optical imaging-monitored, intratumoral RFA-associated RFH-enhanced oncolytic immunotherapy. The interventional oncologic approach enables local delivery of high-dose oncolytic therapeutics into the target tumor, while simultaneously applying intratumoral RFA-associated RF thermal energy to further enhance the tumoricidal effects. This combinatorial local-regional approach can potentially minimize the toxicity caused by the systemic administration of therapeutics, thereby improving the safety profile of the therapy in patients.

LTX-401, as an advanced oncolytic peptide, kill the tumor cells by liberating tumor antigens and immunomodulatory components to prime tumor-specific T cells, thereby promoting antitumor immunity (14, 34). However, LTX-401 is active against normal endothelial cells, keratinocytes and fibroblasts, and also displays a low hemolytic activity (16). In present study, we attempted to fully apply the advantage of image-guided minimally-invasive interventional oncologic technology, by using the new multi-functional perfusion-thermal RF electrode to directly infuse LTX-401 into tumors only. This innovative approach can maximally avoid the effects of LTX-401 on normal tissues and cells, with its further enhanced antitumor effect by intratumoral RFH.

The CD8 molecule is a marker for cytotoxic T cell population (35). CD80 can be found on the surface of various immune cells, including B-cells, monocytes, or T-cells, but most typically at antigen-presenting cells such as dendritic cells (36, 37). In the present study, there is a significantly higher quantity of CD8 positive cells and CD80 positive cells in the combination therapy group, in comparison to other monotherapy groups. These results indicate the involvement of active antigen-presenting activity and cytotoxic T lymphocytes in the combination therapy group, while RFH can further promote the anticancer effect of LTX-401 through the ICD pathway. Thus, since both primary and secondary malignancies often generate resistance to chemotherapies, LTX-401 may become an effective alternative to traditional chemotherapy.

In the present study, we also successfully validated the technical feasibility using our recently established ICG-based interventional optical imaging to assess the response of hepatic tumors to the RFH-enhanced intratumoral oncolytic immunotherapy. In plasma, ICG has an absorption peak around 807 nm and an emission peak around 822 nm, which well suited in the requirement of the new interventional OI system at its first near-infrared (NIR-I) window (32, 33). To apply our new interventional OI technique in monitoring of RFH-enhanced oncolytic immunotherapy, we first optimized ICG dose and detection time-window for intracellular uptake by VX2 tumor cells, with conclusion of the optimized ICG concentration at 100 μg/mL and optimized time window for the best detection of ICG at 24 hours after the ICG treatment. We then further successfully validated the capability of the interventional OI system to evaluate tumor viability in *in-vitro* and *in-vivo*, which were subsequently confirmed using the “gold standard” optical/X-ray imaging and different pathologic/laboratory examinations. The results from our present study showed that this new interventional OI approach enables us to not only detect deep-sited tumors, but also allows for assessment of tumor vitality *in-vivo*, which may provide an excellent advantage for intraprocedural instant detection of residual tumor and the need for additional ablation at the time of the treatment to completely eradicate tumor.

## Conclusion

We present a new interventional oncologic technique, interventional OI- monitored synergistic effects of intratumoral RFH-enhanced oncolytic immunotherapy. The approach may open new avenues to effectively manage those patients suffering from larger and irregular unresectable malignancies, not only in liver but also possibly in other organs.

## Data Availability Statement

The original contributions presented in the study are included in the article/supplementary material. Further inquiries can be directed to the corresponding author.

## Ethics Statement

The animal study was reviewed and approved by Institutional Animal Care and Use Committee of University of Washington.

## Author Contributions

The conception and design of the study: XY. The acquisition of data: HZ and FZ. The analysis and interpretation of data: HZ, FZ, HJ, and XY. Drafting the article and revising it critically for important intellectual content: All authors. All authors contributed to the article and approved the submitted version.

## Funding

This study was supported by the NIH R01EB028095 grant.

## Conflict of Interest

The authors declare that the research was conducted in the absence of any commercial or financial relationships that could be construed as a potential conflict of interest.

## Publisher’s Note

All claims expressed in this article are solely those of the authors and do not necessarily represent those of their affiliated organizations, or those of the publisher, the editors and the reviewers. Any product that may be evaluated in this article, or claim that may be made by its manufacturer, is not guaranteed or endorsed by the publisher.
